# Reliability of the global cortical atrophy visual rating scale applied to computed tomography versus magnetic resonance imaging scans in acute stroke

**DOI:** 10.1007/s10072-023-07113-z

**Published:** 2023-11-01

**Authors:** Georgina Hobden, Emma Colbourne, Sarah T. Pendlebury, Nele Demeyere

**Affiliations:** 1 Department of Experimental Psychology, University of Oxford, Oxford, UK; 2 Nuffield Department of Clinical Neurosciences, Wolfson Centre for Prevention of Stroke and Dementia, University of Oxford, Oxford, UK; 3 Departments of General (Internal) Medicine and Geratology, NIHR Oxford Biomedical Research Centre, John Radcliffe Hospital, Oxford, UK

**Keywords:** Stroke, Neuroimaging, Atrophy, Visual rating, Reliability

## Abstract

**Introduction:**

Research using magnetic resonance imaging (MRI) suggests regional cerebral atrophy measures (e.g., frontal lobe, temporal lobe) may predict post-stroke outcomes. Clinical CT scans have excellent potential for use in research but it is unclear whether regional atrophy measures from CT are reliable compared to MRI reference standards.

**Methods:**

We used the Global Cortical Atrophy (GCA) scale to investigate reliability of atrophy measures on CT versus MRI scans from stroke patients originally recruited to the Oxford Cognitive Screening programme. Two raters provided standardised visual ratings at two timepoints. Weighted Kappa statistics assessed the reliability of regional atrophy scores. Spearman’s correlation and a two-way repeated measures ANOVA assessed the reliability of the total score.

**Results:**

On clinically acquired neuroimaging from 98 stroke patients (mean/SD age = 70.97/11.99, 42 female, 84 ischaemic stroke), regional GCA scores on CT versus MRI showed fair to almost perfect intra-rater agreement (*κ* = .50–.87), substantial to almost perfect intra-rater agreement on CT (*κ* = .67–.88), and moderate to almost perfect intra-rater reliability on MRI (*κ* = .50–.89). Regional GCA scores showed mostly moderate to substantial inter-rater reliability on both CT and MRI (*κ* = .43–.69), except the temporal horns and parieto-occipital region. There was a strong correlation between total GCA scores on CT and MRI (*r* (96) = .87–.88, *p* < .001).

**Conclusions:**

These results support the use of cerebral atrophy measures from CT in clinical research, as visual ratings showed generally good agreement between CT and MRI, between raters, and between timepoints.

## Introduction

Magnetic resonance imaging (MRI) is the favoured imaging modality in research studies due to its higher spatial resolution and improved soft tissue contrast, compared to computed tomography (CT; [21]. However, CT is the standard imaging method in many clinical settings, as it offers substantial time and cost advantages, and it is better tolerated by acutely unwell patients, such as patients with stroke [21].

The large numbers of routinely performed clinical CT scans have great potential for use in research studies that seek to understand the relationship between neuroimaging factors and clinical outcomes in patient populations [8]. Previous studies in stroke patients have employed routinely acquired CT for lesion-symptom mapping [13] and clinical risk prediction [16, 20], and increasing research is focused on the relationship between CT-derived measures of cerebral atrophy and post-stroke outcomes [1].

The relationship between CT-derived cerebral atrophy measures and post-stroke outcomes has mostly been assessed using *global* cerebral atrophy measures [1]. However, evidence from other imaging modalities—including MRI, diffusion-weighted imaging (DWI), and positron emission tomography (PET)—indicates that *regional* cerebral atrophy may better predict important post-stroke outcomes, such as performance on the Mini Mental State Examination [11] and development of post-stroke dementia [22]. Determining whether these findings can be replicated using routinely performed clinical CT scans is of key clinical and academic interest, as capitalising on the large numbers of routinely acquired CT scans would allow for more sensitive studies in larger and more representative patient samples.

So far, to our knowledge, it has not been established whether regional cerebral atrophy measures can be reliably derived from CT and whether these are comparable to MRI reference standards. Previous studies have reported moderate to excellent agreement between visual ratings of global atrophy, deep atrophy, superficial atrophy, and medial temporal lobe atrophy on CT versus MRI [8, 18, 19]. However, it remains uncertain whether reliable estimates of cerebral atrophy in key brain regions—such as the frontal lobe, parieto-occipital lobe, and temporal horns—can be derived from routinely acquired CT imaging.

The present study therefore sought to determine using clinically acquired paired CT and MRI scans from stroke patients: (i) the agreement between CT versus MRI ratings of regional cerebral atrophy, and (ii) intra- and inter-rater agreement for regional cerebral atrophy measures on CT and MRI. We also sought to confirm the correlation between total cerebral atrophy measures on CT versus MRI shown by previous research (e.g., [8].

## Methods

### Participants

This study analysed data collected within the Oxford Cognitive Screening (OCS) programme [6, 7], which recruited a consecutive sample of stroke survivors during acute hospitalisation, assessed domain-specific cognition, and collected routinely acquired clinical brain imaging. Participants were recruited for the OCS programme from Oxford University Hospital’s acute stroke unit between 2016 and 2020. The OCS programme included all patients with a confirmed diagnosis of stroke, who were at least 18 years of age, were able to remain alert for 20 min, and were able to provide informed consent.

The present investigation included all patients recruited for the OCS programme who had undergone both routine clinical CT and MRI brain imaging during the acute/sub-acute stage of stroke (i.e., within 4 weeks post-stroke). A total of 98 patients met these inclusion criteria. Table 1 presents demographic and clinical details of the resultant sample of 98 stroke patients, as recorded by relevant medical records.

**Table 1:** Patient demographic and clinical information

Characteristics	Statistics
Age, *M (SD)*	70.97 (11.99)
Education, *M (SD)*	12.54 (3.01)
NIHSS, *M (SD)*	4.82 (4.13)
Sex, *N (%)*	Female: 42 (42.86); Male: 56 (57.14)
Handedness, *N (%)*	Right: 65 (66.33); Left: 3 (3.06); Unknown: 30 (30.61)
Stroke type, *N (%)*	Ischaemic: 84 (85.71); Haemorrhagic: 4 (4.08); Ischaemic and haemorrhagic: 2 (2.04); Unknown: 8 (8.16)
Stroke lateralisation, *N (%)*	Unilateral right hemisphere: 41 (41.84); Unilateral left hemisphere: 34 (34.69); Bilateral: 14 (14.29); Unknown: 9 (9.18)
Stroke vasculature, *N (%)*	Anterior cerebral artery: 3 (3.06); Middle cerebral artery: 36 (36.73); Posterior cerebral artery: 10 (10.20); Superior cerebellar artery: 14 (14.29); Posterior inferior cerebellar artery: 6 (6.12); Basilar artery: 5 (5.10); Multiple territories: 15 (15.31); Unknown: 9 (9.18)

*NIHSS* National Institutes of Health Stroke Scale*, M mean, SD standard deviation*

### Brain imaging

CT scans were non-enhanced (slice thickness 5 mm) and MRI scans were either T1- or T2-weighted, depending on clinical availability. If a patient had both T1- and T2-MRI available, we analysed the highest quality scan (i.e., scan with least imaging artefacts and noise).

### Visual rating of regional cerebral atrophy

Two experienced neuroimaging researchers (GH and EC) visually rated cerebral atrophy on axial CT and MRI head scans independently, while blinded to demographic and diagnostic information. Both raters were also blind to MRI visual ratings at the time of CT analysis and vice versa. Both raters performed visual ratings twice on the complete imaging dataset at an interval of at least 2 weeks. Both raters were blind to previous ratings during the second rating session.

For visual ratings, both raters used the Global Cortical Atrophy (GCA) scale, first devised by Pasquier et al. [14]. This scale evaluates cerebral atrophy in 13 brain regions, with most regions evaluated separately in each hemisphere (Table 2). The scale rates atrophy in each region on a 0–3 scale, with scores corresponding to absent (0), mild (1), moderate (2), and severe (3) atrophy [14]. If a region was severely affected by a stroke lesion so that cerebral atrophy could not be evaluated with confidence, the affected region scored the same as the anatomically homologous region in the opposite hemisphere. We also calculated a measure of total cerebral atrophy by summing the scores from all regions (range = 0–39) [14].

**Table 2:** Regions used to evaluate cerebral atrophy in the present study. These regions were originally outlined in the Global Cortical Atrophy scale [14]. In line with criteria outlined by Pasquier et al., each region was allocated a score between 0–3, where scores reflected the following: 0 (absent), 1 (mild), 2 (moderate), severe (3)

Region		Hemisphere
Sulcal dilatation	Frontal	Left
		Right
	Parieto-occipital	Left
		Right
	Temporal	Left
		Right
Ventricular dilatation	Frontal horns	Left
		Right
	Occipital horns	Left
		Right
	Temporal horns	Left
		Right
	Third ventricle	Central

### Statistical analysis

We used weighted Kappa values with linear weighting to determine the intra-rater, inter-rater, and inter-modality (CT versus MRI) reliability of regional atrophy ratings. Agreement was interpreted according to Landis and Koch: < 0 = less than chance agreement; 0.01–0.20 = slight agreement; 0.21–40 = fair agreement; 0.41–0.60 = moderate agreement; 0.61–0.80 = substantial agreement; 0.81–0.99 almost perfect agreement [10].

In addition, we investigated the agreement between total GCA scores on CT versus MRI by performing a Spearman correlation. We also used a 2 raters × 2 imaging modalities repeated measures ANOVA to investigate differences between CT and MRI ratings for the paired scans by the two raters. As both raters conducted CT and MRI ratings at two timepoints, we analysed CT-MRI agreement using ratings from the first timepoint only.

### Data availability

All visual ratings used in the analyses of the present study are openly available on the Open Science Framework (https://osf.io/y8q7n/). CT and MRI scans cannot be shared openly, due to research governance restrictions on the use of clinical imaging data.

## Results

CT scans were performed on average 0.49 days after hospitalisation for stroke (*SD* = 1.33, range = 0–7). MRI scans were performed on average 2.61 days after stroke (*SD* = 3.44, range = 0–26). CT and MRI scans were separated by on average 2.31 days (*SD* = 3.33, range = 0–26). Of the 98 paired CT and MRI scans, CT was obtained before, after, and on the same day as MRI in 75, 4, and 19 cases, respectively. Of the 98 MRI scans, 30 scans were T1-weighted and 68 scans were T2-weighted.

### CT versus MRI

**Fig. 1:**
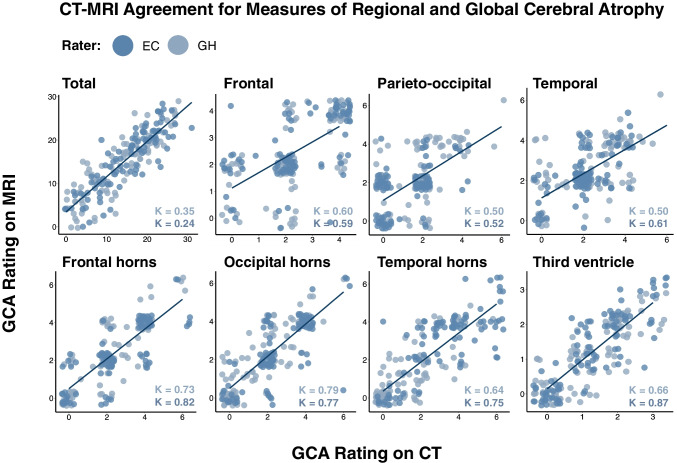
CT-MRI agreement for regional and total cerebral atrophy measures. Each region is plotted individually. Datapoints from each rater are plotted in different colours. Weighted Kappa values are provided in the lower right-hand corner of each plot (font colour corresponds to rater)

**Fig. 2:**
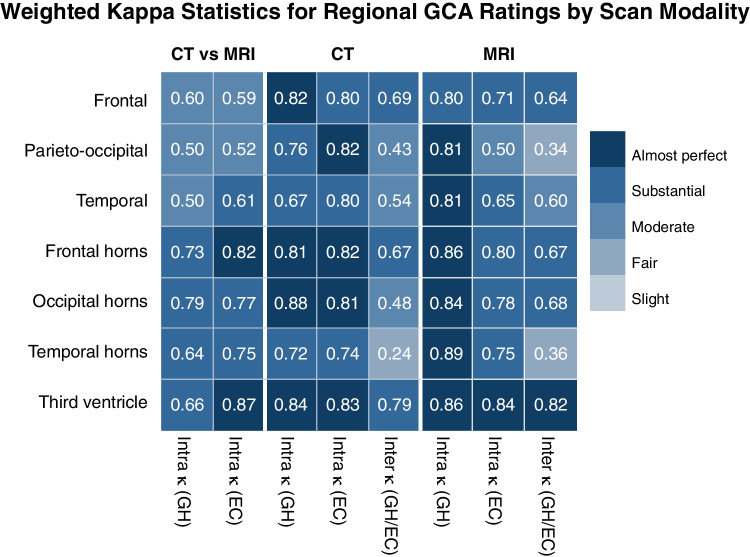
Weighted Kappa statistics for regional cerebral atrophy measures. Weighted Kappa statistics are shown for intra-rater reliability on CT versus MRI, intra- and inter-rater reliability on CT, and intra- and inter-rater reliability on MRI. Colour coding corresponds to Landis and Koch interpretations of weighted Kappa (see “Methods”)

Weighted Kappas indicated moderate to substantial agreement between regional measures of sulcal atrophy on CT and MRI for both raters (*κ* = 0.50–0.61). CT-MRI agreement was substantial to almost perfect for regional measures of ventricular atrophy (*κ* = 0.64–0.87). When intra-rater reliability was averaged across raters, the highest CT-MRI agreement was for the frontal horns and occipital horns, and the lowest CT-MRI agreement was for the parieto-occipital region. Finally, the total cerebral atrophy measure showed fair agreement between CT and MRI for both raters (*κ* = 0.24–0.35). CT-MRI ratings are plotted by region in Fig. 1. Figure 2 provides all CT-MRI weighted Kappa values by region.

**Fig. 3:**
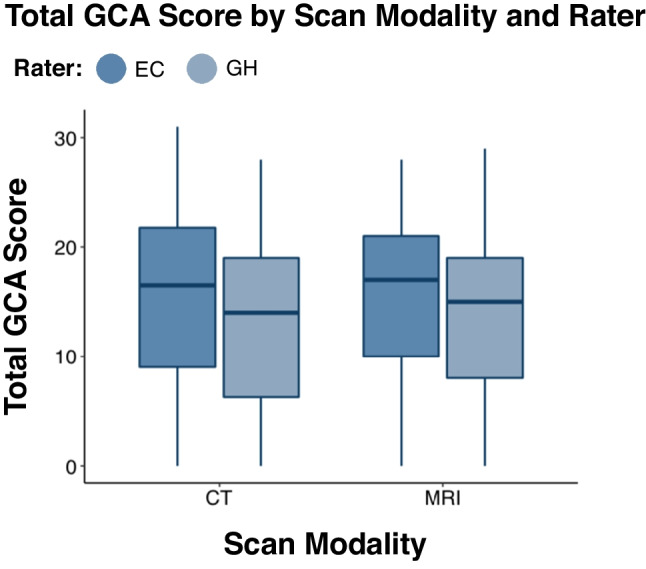
Total atrophy scores plotted according to scan modality and rater. Total atrophy scores were consistently higher on CT versus MRI. Total atrophy was rated consistently higher by EC compared to GH

We further investigated CT-MRI agreement for the total GCA score using Spearman’s correlation and a two-way repeated measures ANOVA with scan modality (CT versus MRI) and rater (GH versus EC) as within-subject variables and total GCA score as the dependent variable. There was a strong correlation between total GCA scores on CT and MRI (*r* (96) = 0.87, *p* < 0.001 for both raters). The two-way repeated measure ANOVA showed there was a significant difference in total GCA score depending on scan modality (*F* (1, 97) = 6.52, *p* < 0.001), but the effect size was small (*η*^*2*^_*G*_ = 0.003). The total GCA score was significantly lower (i.e., atrophy was quantified as less severe) for CT scans (*M* = 14.13, *SD* = 10.39) than for MRI scans (*M* = 14.94, *SD* = 9.63). There was also a significant difference in total GCA score depending on rater (*F* (1, 97) = 46.96, *p* < 0.001), but the effect size was also small (*η*^*2*^_*G*_ = 0.017). Total GCA scores from GH (*M* = 13.58, *SD* = 10.28) were lower than total GCA scores from EC (*M* = 15.49, *SD* = 9.63) (Fig. 3).

### CT versus CT (intra-rater)

Intra-rater agreements for regional measures of sulcal atrophy on CT were in the range *κ* = 0.67–0.82 for both raters, indicating substantial to almost perfect agreement for all sulcal regions. Intra-rater agreement for regional measures of ventricular atrophy on CT was also substantial to almost perfect for both raters (*κ* = 0.72–0.88). When intra-rater reliability was averaged across raters, the highest intra-rater agreement was for the occipital horns, and the lowest intra-rater agreement was for the temporal horns. Intra-rater agreement for the global cerebral atrophy measure was substantial for both raters (*κ* = 0.62–0.69) (Fig. 2).

### CT versus CT (inter-rater)

Inter-rater agreements for regional measures of sulcal atrophy on CT were in the range *κ* = 0.43–0.69, indicating moderate to substantial inter-rater agreement for all sulcal regions. Inter-rater agreement for regional measures of ventricular atrophy on CT was fair to substantial for all regions (*κ* = 0.24–0.79). The highest inter-rater agreement was for the third ventricle (*κ* = 0.79), and the lowest inter-rater agreement was for the temporal horns (*κ* = 0.24). Inter-rater agreement for the total cerebral atrophy measure on CT was fair (*κ* = 0.35) (Fig. 2).

### MRI versus MRI (intra-rater)

Intra-rater agreements for regional measures of sulcal atrophy on MRI were in the range *κ* = 0.50–0.81 for both raters, indicating moderate to almost perfect agreement for all sulcal regions. Intra-rater agreement for regional measures of ventricular atrophy on MRI was substantial to almost perfect for both raters (*κ* = 0.75–0.89). When intra-rater reliability was averaged across raters, the highest intra-rater agreement was for the third ventricle and the lowest intra-rater agreement was for the parieto-occipital region. Intra-rater agreement for the total cerebral atrophy measure was moderate to substantial for both raters (*κ* = 0.47–0.65) (Fig. 2).

### MRI versus MRI (inter-rater)

Inter-rater agreements for regional measures of sulcal atrophy on MRI were in the range *κ* = 0.34–0.64, indicating fair to substantial inter-rater agreement for all sulcal regions. Inter-rater agreement for regional measures of ventricular atrophy on MRI was fair to almost perfect for all regions (*κ* = 0.36–0.82). The highest inter-rater agreement was for the third ventricle (*κ* = 0.82) and the lowest inter-rater agreement was for the parieto-occipital region (*κ* = 0.34). Inter-rater agreement for the total cerebral atrophy measure on MRI was fair (*κ* = 0.24) (Fig. 2).

## Discussion

Using paired clinically acquired CT and MRI scans from the acute/subacute stage post-stroke, the present study found that standardised visual ratings of regional cerebral atrophy showed generally good agreement between CT and MRI, between raters, and between timepoints. This is the largest study to date to our knowledge examining CT-MRI agreement for visual ratings of cerebral atrophy and the first study to demonstrate the intra- and inter-rater reliability of fine-grained regional atrophy measures on CT. By demonstrating that atrophy measures from CT are comparable to those from MRI, and that intra- and inter-rater reliability on CT- versus MRI-derived visual ratings are similar, our findings support the use of routinely acquired CT imaging for clinical research studies investigating the relationship between cerebral atrophy and clinical outcomes in patient populations.

First, regional measures of cerebral atrophy showed moderate to almost perfect agreement on CT and MRI. This extends the results of previous studies that investigated CT-MRI agreement for measures of global cerebral atrophy using less fine-grained visual rating scales. Wattjes et al. [19] reported excellent intra-rater agreement between measures of global cerebral atrophy on CT and MRI in a sample of memory clinic patients (*n* = 30, mean weighted Kappa = 0.83). Similarly, Ferguson et al. [8] reported moderate-to-substantial intra-rater agreement between CT and MRI for measures of superficial atrophy and deep atrophy in a sample of stroke patients (*n* = 70). While global cerebral atrophy has been shown to predict important post-stroke clinical outcomes, such as futile recanalisation [12], future studies may benefit from a more detailed region-specific approach to atrophy assessment, particularly studies that seek to better understand risk factors for cognitive impairment after stroke [11, 15, 22]. The present study provides a fundamental basis for future research by demonstrating that regional atrophy measures on CT are broadly comparable to those derived from higher resolution MRI, and thus suitable for use in clinical research.

Although CT-MRI agreement was moderate to almost perfect for regional atrophy measures, agreement was lower for total atrophy measures. Nevertheless, we observed a strong correlation between total GCA ratings on CT and MRI, which suggests that the lower CT-MRI agreement for the total cerebral atrophy ratings may have been driven by systematic differences in visual ratings on CT versus MRI. This is supported by the results of the two-way repeated measures ANOVA, which showed a trend towards higher total atrophy scores on MRI versus CT for both raters. Although the cause of this systematic difference in scoring is not entirely clear, it is possible that atrophy appeared superficially worse on MRI because of its higher resolution compared to CT [21]. Alternatively, MRI may be more sensitive to atrophy in certain brain regions - for example, the temporal lobe, which is frequently obscured by hyperintense bone on CT imaging. Higher scores on MRI may stem from such increased regional sensitivities, as the total GCA score in the present study was calculated by summation of regional scores.

The present study demonstrated mostly substantial to almost perfect intra- and inter-rater reliability for regional cerebral atrophy ratings on CT. The only exception to this was inter-rater reliability for temporal horn atrophy on CT. Nevertheless, intra-rater reliability for temporal horn atrophy on CT was still substantial, which suggests that the rating scale was applied differently by the two trained raters for this brain region. Therefore, the relatively low inter-rater reliability for temporal horn atrophy could be mitigated in future studies by providing more specific operational definitions and/or exemplars for each temporal horn atrophy score. However, as stated earlier, temporal horn evaluation may be impacted by the presence of hyperintense bone effects. Therefore, although the GCA scale was specifically designed for application on axial slices, future studies may obtain more reliable assessments of temporal horn atrophy on coronal CT slices.

We found lower CT-MRI agreement and lower inter-rater agreement on CT for measures of total cerebral atrophy, compared to previous studies that investigated the reliability of total cerebral atrophy measures on CT and MRI [8, 19]. This is likely due to several differences between our study and previous studies. Firstly, our measurement of total cerebral atrophy included a larger range of available scores (range = 0–39) than previous studies (e.g., range = 0–3; [19]. As the magnitude of Kappa is influenced by the number of categories in the measurement scale, this is likely to have affected our Kappa values [17]. Secondly, our measure of total atrophy may have been impacted by the presence of acute stroke lesions. Because we allocated any region obscured by stroke the same score as the homologous region in the opposite hemisphere, any small discrepancies in regional scoring may have inflated differences in the total measure. Despite these differences, however, our analysis still demonstrated fair CT-MRI agreement and inter-rater agreement for total atrophy measures.

Our findings support the use of regional cerebral atrophy measures from CT for future research in patient populations. CT is the most common imaging modality in acute hospital admissions for stroke, so there is great untapped potential for these scans to be used in research studies investigating the relationship between CT-derived neuroimaging measures and post-stroke outcomes. A particularly interesting avenue for future research is the relationship between regional cerebral atrophy and domain-specific cognitive functioning after stroke, given the clearly demonstrated association between both temporal lobe atrophy and memory, and between frontal lobe atrophy and executive impairments [4]. Furthermore, as localised forms of cerebral atrophy radiologically characterise various dementias [2, 3, 5, 9], future studies should investigate the association between CT-derived regional cerebral atrophy measures and post-stroke dementia. If CT-derived regional atrophy measures are reliably associated with post-stroke cognitive outcomes, cerebral atrophy measures could be incorporated into clinical risk prediction algorithms to help identify patients at risk of post-stroke cognitive impairment and/or post-stroke dementia. This would be particularly valuable should automated atrophy assessment tools become widely available for CT imaging.

There were some limitations in the present study. Firstly, as we used routine clinically acquired CT and MRI brain scans, these often included mild motion artefacts and suboptimal axial slice angulation. This may explain some of the discrepancy in regional cerebral atrophy measures on CT and MRI. Nevertheless, demonstrating the reliability of the GCA scale in a clinically representative sample with ‘imperfect’ brain scans adds substantially to the clinical relevance of the present study. Secondly, we analysed only T1- and T2-MRI so it is not clear whether CT-derived measures of regional cerebral atrophy agree equally well with measures from other MRI sequences, such as fluid attenuated inversion recovery sequences.

Overall, the present study demonstrated that regional cerebral atrophy measures show good agreement between CT and MRI. Furthermore, intra- and inter-rater agreements for regional atrophy measures from CT were broadly compared to measures from MRI. The present study therefore supports the use of routinely acquired CT brain imaging in research, as utilising clinically acquired CT scans will enable important clinical questions to be investigated in highly representative patient samples.
